# Rare, but potentially risky, high individual increase of self-reported sexual arousal in men, who have committed sexual offenses against children, while being confronted with experimental sexual stimuli — a retrospective data analysis

**DOI:** 10.1038/s41443-023-00802-5

**Published:** 2023-12-12

**Authors:** Kirsten Jordan, Peter Fromberger, Isabel Müller, Tamara Sheila Nadine Wild, Jürgen Leo Müller

**Affiliations:** 1 Department of Psychiatry and Psychotherapy, Forensic Psychiatry and Psychotherapy, University Medical Center, University of Goettingen, Goettingen, Germany; 2 University of Goettingen, Goettingen, Germany; 3 Asklepios Clinic for Forensic Psychiatry and Psychotherapy, Goettingen, Germany

**Keywords:** Preclinical research, Human behaviour

## Abstract

This data analysis was initiated to further understand the infrequent yet intense instances of sexual arousal and signs of decompensation that emerge after exposing men who have committed sexual offenses against children to experimental sexual stimuli. We analyzed retrospectively and exploratory data of a self-developed sexual arousal questionnaire (“Current-State-of-Emotions-Questionnaire”, CSEQ) with the following objectives: (i) examine subjective sexual arousal changes elicited during confrontation with experimental sexual stimuli, (ii) analyze these sexual arousal changes at an individual level to detect large responses, and (iii) ask for associations between large responses in sexual arousal and individual characteristics of participants, e.g., demographic, clinical, and criminological parameters. The sample consisted of 241 adult, male Germans, comprising four groups: Ten individuals who have committed sexual offenses against children and have been placed in forensic psychiatric facilities (ISOCFP), 31 non-hospitalized individuals with sexual interest in children (ISIC), eight individuals who have committed other offenses and have been placed in forensic psychiatric facilities (IOFP), and 192 individuals without sexual interest in children and offense histories (IWO). We found a significant increase in subjective *sexual arousal* after confrontation with sexual stimuli (three experiments: initial orientation*: Z* = −4.819*, p* < .001, sexual distractor task: *Z* = −2.954*, p* = .003, stimulus rating: *Z* = −6.899*, p* < .001). Up to 14.3% of participants indicated high sexual arousal values before, but mainly after the experiments, with 20.0% of ISOCFP, 12.9% of ISIC, 12.5% of IOFP, and 14.6% of IWO. ISOCFP and ISIC with high sexual arousal were more likely to be diagnosed with paraphilia (pedophilia) and/or affective disorders, and to receive anti-depressive treatment. We assume a higher emotional lability or/and reduced emotion regulation abilities in those individuals. A careful weighing up of anticipated scientific knowledge gain and a potentially, though rare, increased risk of recidivism or decompensation seems indispensable.

## Introduction

### An occasional study observation – the motivation of the current data analysis

This data analysis was initiated to further understand the infrequent yet intense instances of sexual arousal and signs of decompensation that emerge after exposing men who have committed sexual offenses against children to experimental sexual stimuli. Throughout our series of experiments aimed at gauging sexual interest and orientation through eye movements, we noticed these uncommon yet marked episodes of sexual arousal and signs of decompensation in some participants post-exposure to the experimental stimuli. Given these results, we feel it is crucial to make researchers aware of the potential issues that could arise from presenting sexual stimuli.

For several years, we have been working on research projects aiming at the development of supportive instruments for diagnosis and characterization of people with a sexual interest in children (e.g., [1–5]). To attain this objective, we use different experimental approaches while confronting participants with sexual images. In order to explore the impact of sexual images on our study participants, we regularly ask them about their current feelings, including sexual arousal, immediately before and after each experiment. To this end, we use oral post-experimental debriefings and a self-developed questionnaire (“Current-State-of-Emotions-Questionnaire”, CSEQ, [6, 7]). During the course of the project, we observed that few participants reported an unexpected strong increase of subjective sexual arousal before, but mainly after each experiment. Furthermore, despite strict inclusion and exclusion criteria, one participant from the group of individuals who have committed sexual offenses against children and have been placed in forensic psychiatric facilities (ISOCFP) showed signs of greatly increased emotional lability and decompensation on the ward following study participation. His individual examination was stopped in close coordination with his therapists. Obviously, individual differences, including extreme responses of increased subjective sexual arousal after the confrontation with sexual images, are not surprising. However, in our case, this seems to be of significance, as due to the main objective of our research, the participants of our research comprise also men who have committed sexual offenses. It cannot be excluded that the confrontation with sexual images in our experiments could have increased the risk for (sexual) recidivism or decompensation in these few participants with high sexual arousal [8]. This raises challenging legal, ethical, and clinical conflicts. In order to better understand these occasional observations in our participants, we conducted an exploratory, retrospective data analysis of our self-developed CSEQ [6, 7] for all participants since the beginning of the project.

### Sexual arousal as a potential risk factor for criminal behavior

As one of the first, Ariely and Loewenstein (2006) found that sexual arousal, induced by self-stimulation, had a strong impact on sexual decision-making [9]. Sexually aroused male college students rated sexual stimuli and activities (e.g., a 12-year-old girl, men, a woman who was sweating, having sex with light on) as more attractive compared to a non-aroused state. They also indicated a higher willingness to engage in morally questionable behavior in order to obtain sexual gratification (e.g., to encourage a date to drink, slip her a drug), and to have sex even after the person they were dating said “no”. Additionally, students reported a higher willingness to engage in unsafe sex [9]. Ariely and Loewenstein (2006) suggested that this increase in motivation to have sex seems to decrease the relative importance of other considerations such as behaving ethically correct toward a potential partner or protecting oneself against sexually transmitted diseases. According to these authors, sexual arousal seems to narrow the focus of motivation, i.e., creating a kind of tunnel-vision where goals other than sexual gratification are deferred. Moreover, Ariely and Loewenstein concluded that their participants seemed to have only limited insight into the impact of sexual arousal on their own judgements and behavior, which could be important for both individual and social decision making [9]. Despite a discussion about underlying mechanisms and specificity, several studies have replicated this effect in healthy men and women (e.g., [10], but see: [11]). Several parameters might modulate this association between sexual arousal and riskier behavior. Higher impulsivity and lower intellectual abilities, for instance, are proposed to be associated with riskier (sexual) behavior [12]. For men with low working memory capacity, an increased skin conductance response to aggressive sexual images was a significant predictor for a later stopping point in a date-rape scenario [13]. Furthermore, Most and colleagues (2007) proposed that pleasant erotic stimuli could elicit a transient “emotion-induced blindness” [14]. Recently, Render and Jansen (2021) showed that subjective sexual arousal can also negatively affect feelings of control. Performance in the so-called intentional binding, an experimental paradigm to measure the degree of consciousness of actions, was impaired after watching erotic films [15].

Previous studies showed that sexual aggressors against women exhibit slightly more physiological rape arousal than participants of control groups (for a metaanalysis see: [16]). Craig et al. (2017) demonstrated that aggressive men experienced a higher level of subjective sexual arousal when being confronted with sexually explicit stimuli [17]. Moreover, in laboratory studies, increased self-reported sexual coercion proclivity has consistently been associated with increased sexual arousal in response to erotic stimuli. This effect seems to be more pronounced in men with poor emotion regulation abilities (for an overview see: [8]). The association between sexual arousal and risky sexual behavior, sexual aggression and offending also plays an important role in several theories of sexual offending. According to the Integrated Theory of Offending, for instance, a deviant sexual arousal belongs to the clinical phenomena which can affect the individual’s risk of committing a sexual offense, and can therefore be seen as a potential risk factor for deviant sexual behavior (for an overview see: [18]).

### Inducing sexual arousal in forensic sex research

Deviant sexual interest has repeatedly been described as one of the most important risk factors for recidivism in individuals who have committed sexual offenses. It belongs to the broader construct of sexual deviance which comprises risk-relevant aspects of sexual functioning and self-regulation, such as deviant sexual interest, hypersexuality, and sexualized coping [19–21]. Studies aimed at the experimental measurement of sexual interest usually confront participants with different sexual stimuli in order to find out the sexually preferred stimulus independent of participants’ subjective responses (e.g., [22–26]). For one thing, a valid and reliable measurement of deviant sexual interest, insensitive to manipulations, could significantly support the clinical diagnostic process. On the other hand, considering the above discussed association between sexual arousal and risky behavior, an increased sexual arousal could potentially increase the risk of subsequent sexual crimes. This raises challenging legal, ethical, and clinical conflicts as the target group in this research field comprises mostly individuals who have committed sexual offenses against children. Obviously, researchers are aware of these potential risks and have developed rules and concepts, e.g., inclusion and exclusion criteria for participants to guarantee public safety. However, based on our above-described observations in few individual participants, we assume that an exploratory data analysis to examine possible associations between heightened, individual sexual arousal and specific demographic, clinical, and criminological parameters could be useful in order to determine individual participants who may potentially be at increased risk for recidivism or decompensation after study participation.

### Aim of the current data analysis

Considering our own aforementioned experiences with the impact of sexual images on individual participants we decided to conduct an retrospective, exploratory data analysis of our self-developed CSEQ [6, 7] with the following objectives: (i) examine general subjective sexual arousal changes elicited during the experiments (ii) analyze these sexual arousal changes at an individual level to recognize large responses, and (iii) ask for associations between large responses in sexual arousal and individual characteristics, such as demographic, clinical, and criminological parameters.

## Participants and methods

### Participants

We analyzed data from different studies and three different types of experiments (please see method section). A detailed overview of demographic, clinical, and criminological characteristics of the whole sample (*N* = 241) is given in Table 1. All participants were adult, male and German. Intelligence was assessed by Wechsler Adult Intelligence Scale [27] or Raven’s Progressive Matrices [28]. With respect to the special participant groups and sensitive research objective (i.e., forensic settings, assessment of sexual interest in children), we did not compute sample size a priori, but attempted to find as many study participants as possible. The sample comprised four groups with: Ten individuals who have committed sexual offenses against children and have been placed in forensic psychiatric facilities (ISOCFP), 31 non-hospitalized individuals with sexual interest in children (ISIC), eight individuals who have committed other offenses and have been placed in forensic psychiatric facilities (IOFP), and 192 individuals without sexual interest in children and offense histories (IWO). For most ISOCFP, IOFP and ISIC sexual orientation was assessed based on the victims’ gender. Sexual orientation for IWO, IOFP and ISIC without victims was assessed with the Kinsey scale [29].

**Table 1:** Detailed characteristics of the subject groups: Demographic, clinical and criminological data.

Number of Participants	ISOCFP *n* = 10	ISIC *n* = 31^a^	IOFP *n* = 8	IWO *n* = 192	Statistics^b^
Demographic data^c^					
Age, years (SD)	39.10 (13.60)	40.35 (9.63)	36.88 (11.52)	24.99 (5.55)	*H*(3) = 72.253, *p* < 0.001
Intelligence, overall mean IQ (SD)	78.20 (14.57)	96.07 (19.24)	90.38 (14.56)	114.99 (13.86)	*H*(3) = 55.930, *p* < 0.001
Hospitalization, month (SD)	145.20 (100.23)	n.a.	111.75 (117.62)	n.a.	*U* = 30.000, *p* = 0.408
Highest level of education attained^d^					
No educational qualification	3 (30.0%)	0 (0.0%)	0 (0.0%)	0 (0.0%)	n. a.
Special education school	1 (10.0%)	0 (0.0%)	1 (12.5%)	0 (0.0%)	*χ*^*2*^(1) = 0.000, *p* = 1.000
Secondary school	3 (30.0%)	9 (29.0%)	5 (62.5%)	0 (0.0%)	*χ*^*2*^(2) = 3.294, *p* = 0.193
Junior high school	3 (30.0%)	9 (29.0%)	1 (12.5%)	12 (6.3%)	*χ*^*2*^(3) = 12.600, *p* = 0.006
High school	0 (0.0%)	13 (41.9%)	1 (12.5%)	173 (90.1%)	*χ*^*2*^(2) = 295.872, *p* < 0.001
University of applied science/university	0 (0.0%)	0 (0.0%)	0 (0.0%)	7 (3.6%)	n. a.
Current relationship status^d^					
Single	9 (90.0%)	14 (45.2%)	7 (87.5)	112 (58.3%)	*χ*^*2*^(3) = 220.535, *p* < 0.001
Partnership	1 (10.0%)	3 (9.7%)	1 (12.5%)	71 (37.0%)	*χ*^*2*^(3) = 189.895, *p* < 0.001
Married	0 (0.0%)	6 (19.4%)	0 (0.0%)	6 (3.1%)	*χ*^*2*^(1) = .000 *p* = 1.000
Divorced/separated	0 (0.0%)	7 (22.6%)	0 (0.0%)	3 (1.6%)	*χ*^*2*^(1) = 1.600 *p* = 0.206
Widowed	0 (0.0%)	1 (3.2%)	0 (0.0%)	0 (0.0%)	n. a.
Sexual orientation^e^					
Heterosexual	3 (30.0%)	25 (80.6%)	7 (100%)	174 (90.6%)	*χ*^*2*^(3) = 383.517, *p* < 0.001
Homosexual	2 (20.0%)	5 (16.1%)	1 (12.5%)	15 (7.8%)	*χ*^*2*^(3) = 21.348, *p* < 0.001
Bisexual	5 (50.0%)	1 (3.2%)	0 (0.0%)	3 (1.6%)	*χ*^*2*^(2) = 2.667, *p* = 0.264
Current medication^f^					
Antidepressants	4 (40.0%)	3 (9.7%)	3 (37.5%)	n.a.	*χ*^*2*^(2) = 200, *p* = 0.905
Sedatives	0 (0.0%)	1 (3.2%)	0 (0.0%)	n.a.	n.a.
Neuroleptics	0 (0.0%)	0 (0.0%)	4 (50.0%)	n.a.	n.a.
Antipsychotics	0 (0.0%)	0 (0.0%)	1 (12.5%)	n.a.	n.a.
Testosterone-lowering medication	2 (20.0%)	0 (0.0%)	0 (0.0%)	n.a.	n.a.
ICD-10 Diagnoses^g^					
Pedophilia (F65.4)	5 (50.0%)	10 (33.3%)	n.a.	n.a.	*χ*^*2*^(1) = .756, *p* = 0.384
Organic, mental disorder (F00-F09)	1 (10.0%)	0 (0.0%)	1 (12.5%)	n.a.	*χ*^*2*^(2) = 3.548, *p* = 0.170
Substance abuse/dependence (F10-F19)	4 (40.0%)	5 (16.7%)	6 (75%)	n.a.	*χ*^*2*^(2) = 10.453, *p* = 0.005
Schizophrenia (F20-F29)	0 (0.0%)	0 (0.0%)	3 (37.5%)	n.a.	*χ*^*2*^(2) = 16.000, *p* < 0.001
Affective disorders (F30-F39)	1 (10.0%)	9 (30.0%)	0 (0.0%)	n.a.	*χ*^*2*^(2) = 4.345, *p* = 0.114
Neurotic disorders (F40-F49)	1 (10.0%)	6 (20.0%)	0 (0.0%)	n.a.	*χ*^*2*^(2) = 2.241, *p* = 0.326
Behavioral disorders with somatic disorders (F50-F59)	1 (10.0%)	4 (13.3%)	1 (12.5%)	n.a.	*χ*^*2*^(2) = .076, *p* = 0.963
Personality and conduct disorders (F60-F69, excl. F65.4)	5 (50.0%)	12 (40.0%)	7 (87.5%)	n.a.	*χ*^*2*^(2) = 6.033, *p* = 0.197
Intellectual disabilities (F70-F79)	3 (30.0%)	2 (6.7%)	0 (0.0%)	n.a.	*χ*^*2*^(2) = 5.492, *p* = 0.064
Behavioral disorders with onset in childhood (F90-F99)	0 (0.0%)	3 (10.0%)	0 (0.0%)	n.a.	*χ*^*2*^(2) = 1.920, *p* = 0.383
Criminological data^h^					
Previous convictions, mean (SD)	2.10 (1.91)	72 (1.22)	8.13 (7.49)	n.a.	*H*(2) = 14.767 *p* < 0.001
Number of victims, mean (SD)	7.50 (5.26)	9.21 (26.29)	n.a.	n.a.	*U* = 21.000, *p* = 0.003
Age of victims, mean (SD)	8.46 (2.04)	9.49 (3.41)	n.a.	n.a.	*U* = 64.500, *p* = 0.374
SSPI-score, mean (SD)	4.10 (.99)	3.00 (1.00)	n.a.	n.a.	*U* = 29.000, *p* = 0.026
Consumption of child exploitation material	n.a.	26 (83.9%)	n.a.	n.a.	n.a.

(%) percentage within groups are given.

*ISOCFP* Individuals who have committed sexual offenses against children and have been placed in forensic psychiatric facilities, *ISIC* Individuals with a self-reported sexual interest in children without being placed in forensic psychiatric facilities, *IOFP* individuals who have committed other offenses and have been placed in forensic psychiatric facilities, *IWO* individuals without sexual interest in children and without offense histories.

*n* number of subjects, *SD* standard deviation, *n.a* not applicable.

^a^Information about diagnosis was only available for 30 out of 31 ISIC. With respect to sexual interest in children, for one ISIC an ICD-10 F65.4 diagnoses was only suspected. Three ISIC preferred adults but were also sexually interested in children.

^b^Test statistic for education, relationship, sexual orientation, medication, and ICD10-diagnosis: Chi-square test; Test statistic for demographic and criminological data: non- parametric Kruskal–Wallis test or Mann–Whitney-*U*-Test for independent samples.

^c^Demographic data: All participants were adult, male and German. Age: Bonferroni-corrected post-hoc tests: IWO vs. ISIC, *p* < 0.001, IWO vs. ISOCFP *p* = 0.001, IWO vs. IOFP *p* = 0.015. Intelligence: Intelligence was assessed by the Wechsler Adult Intelligence Scale [27] or Raven’s Progressive Matrices [28]. Data for only 29 out of 31 ISIC were available. Bonferroni-corrected post-hoc tests: IWO vs. ISOCFP, ISIC *p* < 0.001, IWO vs. IOFP *p* = 0.002. Hospitalization reflects the overall time duration of the ISOCFP and IOFP in forensic hospitals.

^d^Highest level of education attained and current relationship status were assessed via self-report for all participants.

^e^Sexual orientation: For most ISOCFP, IOFP and ISIC sexual orientation was assessed based on the victims’ gender. Sexual orientation for IWO, IOFP and ISIC without victims was assessed with the Kinsey scale [29], Ratings: 0 or 1: heterosexual, 2 to 4: bisexual, 5 or 6: homosexual.

^f^Current medication: Current medication for ISOCFP and IOFP was assessed from forensic records. One ISOCFP took antidepressants and testosterone-lowering medication, two IOFP took two neuroleptics, and one IOFP took antidepressants and neuroleptic. For ISIC information about current medication was gathered via therapists. For IWO information about medication was not recorded.

^g^ICD-10 diagnoses: Only those ICD-diagnosis are presented, which were assigned for at least one subject. Participants with an F10-F19 ICD-10 diagnosis had been abstinent from substance abuse for at least one month. Participants with an F20–29 ICD-10 diagnosis did not have any acute psychotic episode at least one month prior to testing. For IOFP, the absence of a diagnosis of pedophilia was an inclusion criterion. For IWO ICD- diagnoses were not available. For this group the absence of any psychiatric illness, one of the inclusion criteria, was assessed via self-report.

^h^Criminological data: Note that criminological data for ISIC is based on self-reports. Bonferroni-corrected post-hoc tests for previous convictions: ISIC vs. IOFP *p* < 0.001 Number and age of victims are reported only for ISOCFP (*n* = 10) and ISIC with at least one sexually abused child (*n* = 14). One ISIC reported about 100 victims. Age of victims was available for eight ISOCFP and 13 ISIC. *SSPI* screening scale for pedophilic interest: [44] SSPI-Score could be computed for 10 ISOCFP and 13 ISIC. Information on consumption of child exploitation material was only available for ISIC. For more details, please see section: Participants.

Among ISOCFP, ISIC and IOFP, 15 out of 49 (30.6%) suffer from paraphilia (pedophilia, ICD-10, F65.4, [30]). Since not all participants took part in all three experiments, Table 2 presents the number of participants per group for each experiment. Behavioral and eye tracking data, but not CSEQ-data, were published in several studies [4, 31–40]. All studies received a positive vote by the Ethics Committee of the Medical Faculty of the Georg-August-University of Goettingen, Germany. All participants provided written informed consent before participation. They did not receive any incentives.

**Table 2:** Number of participants (*n*) per group for each experiment.

Experiment	ISOCFP *n*	ISIC *n*	IOFP *n*	IWO *n*
Initial Orientation	10	30	7	38
Sexual Distractor Task	10	28	8	190
Stimulus Rating	10	28	8	192

The “Current-State-of-Emotions-Questionnaire” (CSEQ) was applied immediately before and after each experiment.

*ISOCFP* Individuals who have committed sexual offenses against children and have been placed in forensic psychiatric facilities, *ISIC* Individuals with a self-reported sexual interest in children without being placed in forensic psychiatric facilities, *IOFP* Individuals who have committed other offenses and have been placed in forensic psychiatric facilities, *IWO* Individuals without sexual interest in children and without offense histories.

#### Individuals who have committed sexual offenses against children and have been placed in forensic psychiatric facilities (ISOCFP)

All 10 ISOCFP were recruited at forensic psychiatric hospitals in Bremen and Goettingen, Germany. They had committed sexual offenses against children and were placed in forensic psychiatric facilities for improvement and safeguarding according to §63 of the German Criminal Code. They were sentenced to a forensic psychiatry as they had committed a crime in a state of a severe mental disorder and had therefore been adjudicated as not or diminished criminally responsible in conjunction with a high risk of criminal re-offense. All data was obtained from forensic records.

#### Individuals who have committed other offenses and have been placed in forensic psychiatric facilities (IOFP)

All eight IOFPs were recruited at forensic psychiatric hospitals in Bremen and Goettingen, Germany. They were placed in forensic psychiatric facilities for improvement and safeguarding according to §63 and §64 of the German Criminal Code. They were sentenced to a forensic psychiatry as they had committed a crime in a state of a severe mental disorder and had therefore been adjudicated as not or diminished criminally responsible in conjunction with a high risk of criminal re-offense. Index delicts of this control group comprised homicide, attempted homicide, robbery, attempted rape, predatory theft, and commercial fraud. All data was obtained from forensic records. One patient of this group refused to complete the CSEQ after the initial orientation experiment, and was therefore excluded from the statistical analysis of the CSEQ in this experiment.

#### Individuals with self-reported sexual interest in children without being placed in forensic psychiatric facilities (ISIC)

Thirty-one ISIC, were included in the study. They were recruited from the outpatient preventive treatment project “Prevention of Sexual Abuse” (PsM, 2011–2020, Goettingen, Germany, [41]). The PsM-project was aimed at adults suffering from a sexual interest in children and/or adolescents, including people who fear that they might commit an act of abuse (e.g., because of the presence of sexual fantasies with children/adolescents), people who consume child sexual exploitation material, and individuals who have committed sexual abuse (for details see: [41]). All criminological data was assessed via self-report. Four ISIC dropped out from the PsM-Project before the end of the study, but at different study parts. Therefore, one ISIC was excluded from statistical analysis of the CSEQ in the initial orientation experiment, three others were excluded from the statistical analysis of the CSEQ in the sexual distractor task and the stimulus rating.

#### Individuals without sexual interest in children and without offense histories (IWO)

All information for the 192 participants of this group was obtained by self-report. All participants of this group were recruited using a targeted advertising strategy by placing recruitment notices on bulletin boards in Goettingen, Germany. Most of them were currently enrolled at the university of Goettingen, Germany (182 out of 192). The remaining participants had a secondary school diploma and were employed.

### Inclusion and exclusion criteria

Inclusion criterion for the ISOCFP was a mandatory hospitalization under treatment for a child-abuse offense. ISIC were included, if they reported a sexual interest in children. Inclusion criteria for the IOFP was the absence of a child-abuse offense and mandatory hospitalization under treatment for an offense, or under treatment for addiction. A clinical evaluation was done for each patient. ISOCFP, ISIC and IOFP were selected based on their cognitive capability, and the risk that might be linked with the experiments and the safety regulations of the ward. Only those individuals were asked for participation, who received a positive evaluation by therapists. Additionally, all ISIC had a mandatory interview with their therapist after study participation, to evaluate any emotional irritation, and a potential increased risk for (sexual) criminal behavior. Based on this selection, 42 men, who were placed in forensic hospitals (ISOCFP, IOFP) were asked for participation. Out of them 18 agreed to participate and 24 rejected to be a part of this study. With respect to ISIC, we did not exactly record the number of individuals who were asked and who denied participation. Approximately between 30 and 40% of the asked participants gave their consent to participate in the study. Inclusion criteria for the IWO were the absence of any psychiatric illness and any deviant sexual fantasies or behavior. None of the invited participants of this group had to be excluded for this data analysis. Exclusion criteria for all participants were acute psychotic episodes or substance abuse during the previous month, and incapability or refusal to sign informed consent.

## Methods

**Fig. 1:**
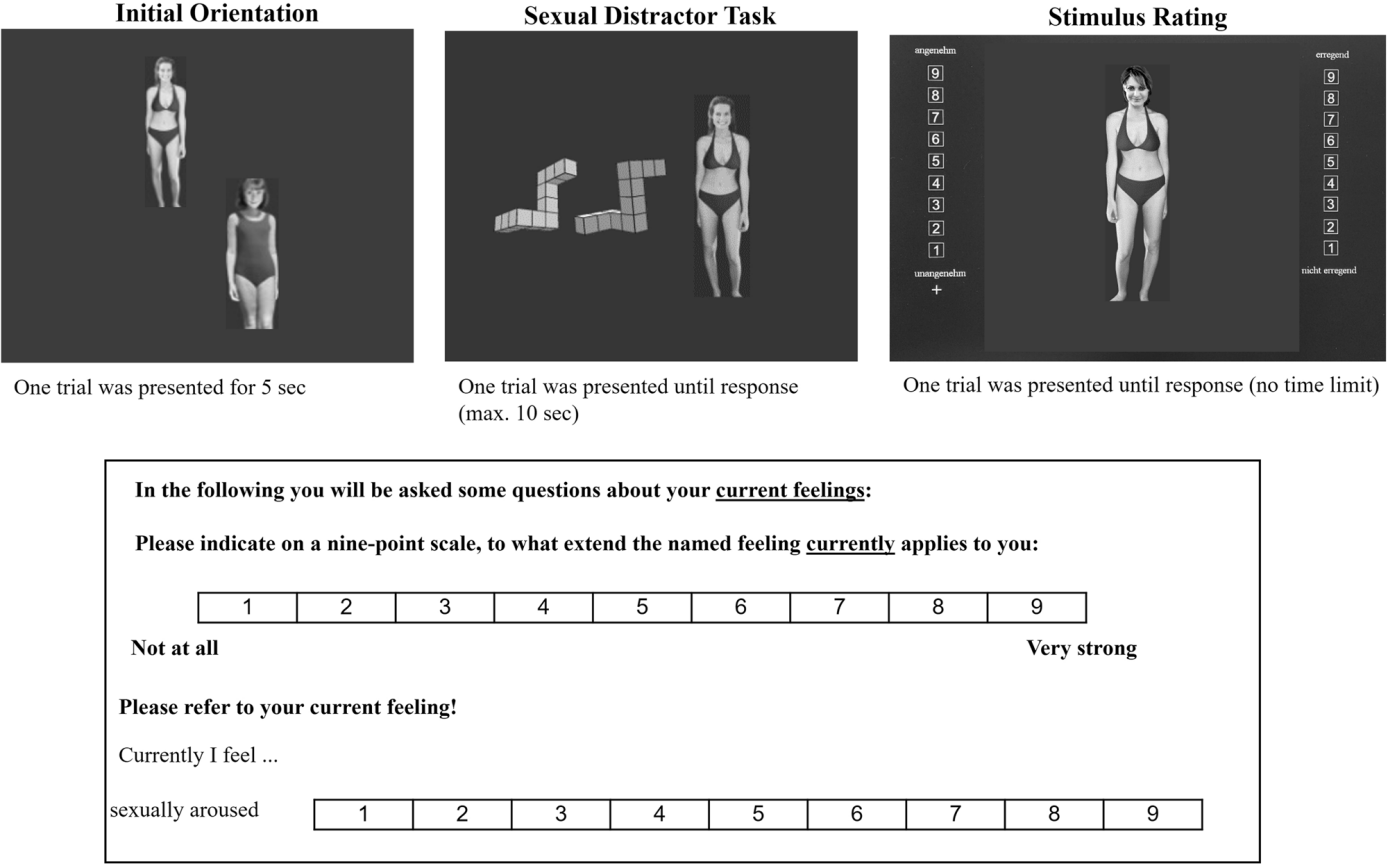
Above: the three experiments: initial orientation, sexual distractor task, stimulus rating. For each experiment a typical training stimulus is presented. Within the experiments, the unclothed version of the stimulus-set was used. Below: Current-State-of-Emotions-Questionnaire (CSEQ). Subject instruction and an example for the dimension *sexual arousal* is given. For details, please see method section.

The self-developed CSEQ was applied directly before and after each experiment in order to detect self-reported changes in emotions due to the experimental procedure ([6, 7], see Fig. 1). We asked participants, to spontaneously rate the extent to which they felt the eight emotions: rage, happiness, disgust, sexual arousal, cheerfulness, anxiety, sadness, and anger on a 9-Point-Likert-scale (min = 1, max = 9). Low scores imply low characteristics, 1 = “not at all”, 9 = “very strong”.

All experiments (Initial Orientation, Sexual Distractor Task, Stimulus Rating) and stimuli are described in detail in several publications (please see [1, 5]). Sexual stimuli in all three types of experiments were taken from the Not-Real-People-picture set [42, 43]. The images are non-pornographic in that they do not contain explicit sexual poses or sexual activity. Figure 1 depicts typical training stimuli, used in each of the experiments. During the whole experimental session, participants were seated in a separate room.

### Data analysis

**Fig. 2:**
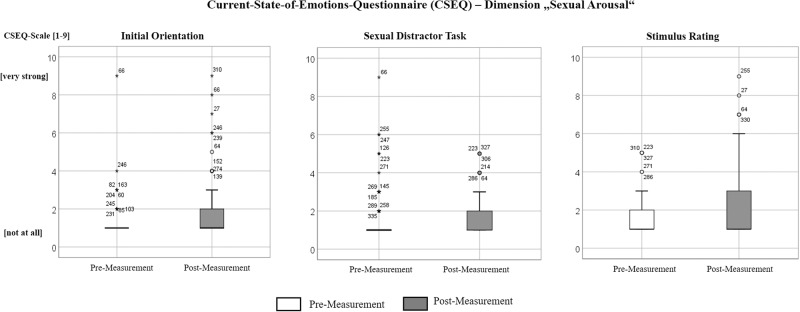
Current-State-of-Emotions-Questionnaire (CSEQ), dimension *sexual arousal*. Box-plots are presented for the whole sample and each experiment. Sample size for each experiment: Initial Orientation Experiment: *N* = 85, Sexual Distractor Task: *N* = 236, Stimulus Rating: *N* = 238.

All statistics were performed with IBM SPSS 26.0 (IBM Corp. and other(s), 2019, NY, USA). In order to focus on the main goal, we only analyzed the dimension *sexual arousal* of the CSEQ. Since most variables were scaled nominally or ordinally, and were not normally distributed, we applied non-parametric statistical tests. For the analysis of pre-post changes, two-tailed Wilcoxon-tests for dependent samples were applied for whole group, each experiment, and within groups. To explore individual data, box-plots were computed. Thereby, high values on the CSEQ were defined as equal to or above 4 on the 9-point Likert scale. The decision for this threshold is based on the data itself. Figure 2 shows, that for five out of six parameters (three experiments each with pre- and post-measurements) values “equal to or above 4” belong to outliers and extreme values. The majority of our participants reported values between “1 and 3”. In order to have a unitary value for all further computations, we decided to choose this rather artificial threshold for our exploratory, retrospective data analysis. We grouped participants with respect to low and high values, independently of experiment or pre-/post-measurement. These subgroups were compared with respect to demographic, criminological, and clinical variables. This was done within each subject group. In dependence of specific data structure, we applied different two-tailed nonparametric tests (Mann–Whitney *U*-test, Chi-square-test). Due to the low number of participants, we also report results only demonstrating a trend.

## Results

### Pre-post changes in the whole sample

After each experiment, a significant increase was found for *sexual arousal (*initial orientation*: Z* = −4.819*, p* < .001, sexual distractor task: *Z* = −2.954*, p* = .003, stimulus rating: *Z* = −6.899*, p* < .001*)* (see Table 3 and Fig. 2). While medians with values of “1” do not change, minimal and maximal values, and box-plot presentations reveal that there are several participants who indicated high values (equal to or above 4 on the 9-Point-Likert-scale), both before but mainly after the experiments.

**Table 3:** The Current-State-of-Emotions-Questionnaire (CSEQ): dimension sexual arousal.

Experiment	Group	Whole Sample *N* = 85	ISOCFP *n* = 10	ISIC *n* = 30	IOFP *n* = 7	IWO *n* = 38
Initial Orientation	Pre-Measurement Median (Min-Max)	1.00 (1–9)	1.00 (1–9)	1.00 (1–3)	1.00 (1–1)	1.00 (1–4)
	Post-Measurement Median (Min-Max)	1.00 (1–9)	1.50 (1–8)	1.50 (1–7)	1.00 (1–9)	1.00 (1–6)
	Statistics^a^	*Z* = −4.819 *p* < 0.001	*Z* = −1.414 *p* = 0.157	*Z* = −3.106 *p* = 0.002	*Z* = −1.633 *p* = 0.102	*Z* = −2.991 *p* = 0.003
	High values in pre-Measurement *n* Ss. (%)^b^	2/85 (2.36%)	1/10 (10.00%)	0/30 (0.00%)	0/7 (0.00%)	1/38 (2.63%)
	High values in post-Measurement *n* Ss. (%)^b^	9/85 (10.59%)	1/10 (10.00%)	3/30 (10.00%)	1/7 (14.28%)	4/38 (10.53%)
		**Whole Sample** ***N* = 236**	**ISOCFP** ***n* = 10**	**ISIC** ***n* = 28**	**IOFP** ***n* = 8**	**IWO** ***n* = 190**
Sexual Distractor Task	Pre-Measurement Median (Min-Max)	1.00 (1–9)	1.00 (1–9)	1.00 (1–2)	1.00 (1–2)	1.00 (1–6)
	Post-Measurement Median (Min-Max)	1.00 (1–5)	1.00 (1–4)	1.00 (1–4)	1.00 (1–3)	1.00 (1–5)
	Statistics^a^	*Z* = −2.954 *p* = 0.003	*Z* = −0.687 *p* = 0.492	*Z* = −1.725 *p* = 0.084	*Z* = −1.00 *p* = 0.317	*Z* = −2.292 *p* = 0.022
	High values in pre-Measurement *n* Ss. (%)^b^	7/236 (2.97%)	1/10 (10.00%)	0/28 (0.00%)	0/8 (0.00%)	6/190 (3.16%)
	High values in post-Measurement *n* Ss. (%)^b^	7/236 (2.97%)	1/10 (10.00%)	1/28 (3.57%)	0/8 (0.00%)	5/190 (2.63%)
		**Whole Sample** ***N* = 238**	**ISOCFP** ***n* = 10**	**ISIC** ***n* = 28**	**IOFP** ***n* = 8**	**IWO** ***n* = 192**
Stimulus rating	Pre-Measurement Median (Min-Max)	1.00 (1–5)	1.00 (1–3)	1.00 (1–5)	1.00 (1–5)	1.00 (1–5)
	Post-Measurement Median (Min-Max)	1.00 (1–9)	1.00 (1–5)	2.00 (1–8)	1.00 (1–6)	1.50 (1–9)
	Statistics^a^	*Z* = −6.899 *p* < 0.001	*Z* = −1.633 *p* = 0.102	*Z* = −2.992 *p* = 0.003	*Z* = −1.732 *p* = 0.083	*Z* = −5.862 *p* < 0.001
	High values in pre-Measurement *n* Ss. (%)^b^	7/238 (2.94%)	0/10 (0.00%)	1/28 (3.57%)	1/8 (12.50%)	5/192 (2.60%)
	High values in post-Measurement *n* Ss. (%)^b^	27/238 (11.34%)	1/10 (10.00%)	3/28 (10.71%)	1/8 (12.50%)	22/192 (11.46%)

Median values (min-max), statistics for the pre- and post-measurement, and individuals with high values, for whole sample, each group and each experiment are presented.

*ISOCFP* Individuals who have committed sexual offenses against children and have been placed in forensic psychiatric facilities, *ISIC* Individuals with a self-reported sexual interest in children without being placed in forensic psychiatric facilities, *IOFP* Individuals who have committed other offenses and have been placed in forensic psychiatric facilities, *IWO* Individuals without sexual interest in children and without offense histories. N/*n* Number of Subjects, *Min* Minimum, *Max* Maximum.

^a^Test Statistics: two-tailed Wilcoxon-tests for dependent samples.

^b^High values in Pre- or Post-Measurement are defined as values equal to or above “4” at the 9-Point-Likert-Scale. Given is absolute number of subjects with values equal to or above “4”, and percentage with respect to the appropriate group.

### Pre-post changes within groups

Pre-post comparisons for the initial orientation experiment yielded a statistically significant increase of subjective *sexual arousal* only for the IWO (*Z* = −2.991, *p* = 0.003) and ISIC (*Z* = −3.106, *p* = 0.002). Looking at the individual level, up to four participants per group (0–14.28%) reported high values (equal to or above 4 on the 9-Point Likert scale) before and/or after the experiment, with relatively most participants in the IOFP (1 out of 7 participants, 14.28%).

Pre-post comparisons for the sexual distractor task yielded a significant increase of experienced *sexual arousal* only for the IWO (*Z* = −2.292, *p* = 0.022). High values were indicated by up to six participants per group (0–10.00%), with relatively most participants in the ISOCFP (1 out of 10 participants, 10.00%).

**Fig. 3:**
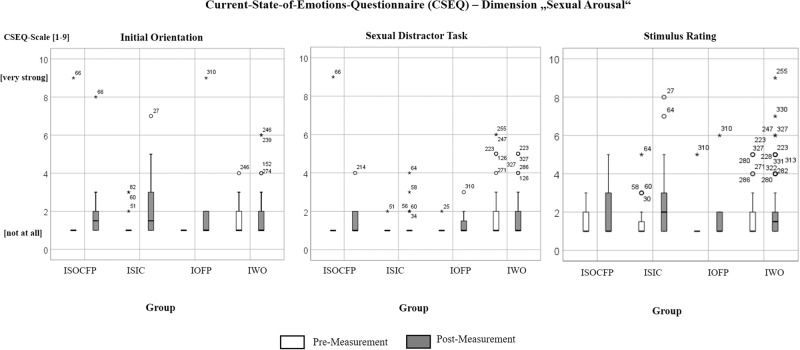
Current-State-of-Emotions-Questionnaire (CSEQ), dimension *sexual arousal*. Box-plots are presented for each group and each experiment. For sample size per group and experiment, please refer to Tables 2 and 3. ISOCFP: Individuals who have committed sexual offenses against children, and have been placed in forensic psychiatric facilities, ISIC: individuals with self-reported sexual interest in children who were not sentenced to forensic psychiatric facilities, IOFP Individuals who have committed other offenses and have been placed in forensic psychiatric facilities, IWO individuals who have not committed any offenses and without a sexual interest in children.

For the stimulus rating, pre-post comparisons yielded a significant increase of subjective *sexual arousal* for the IWO (*Z* = −5.862, *p* < 0.001) and ISIC (*Z* = −2.992, *p* = 0.003). At individual level, up to 22 participants were seen with high values (0–12.50%) with relatively most participants in the IOFP (1 out of 8 participants, 12.50%), (see Fig. 3, Table 3).

### Characteristics of participants with high values in sexual arousal

Overall, 14.52% out of all participants (35 out of 241) indicated subjective arousal levels equal to or above 4 on the 9-point Likert scale (with respect to all experiments and both measurements). Looking at the whole sample, individuals with low and high values of *sexual arousal* did not differ statistically regarding age, intelligence, and current sexual activity (see table S1).

At group level, 20.00% of ISOCFP (2 out of 10), 12.90% of the ISIC (4 out of 31), 12.50% from the IOFP (1 out of 8), and 14.58% from the IWO (28 out of 192) indicated high sexual arousal values. Table 4 presents demographic, clinical, and criminological variables and statistical analyses for each group. Within groups, individuals with low and high values did not differ with respect to age, intelligence, current sexual activity, relationship status, testosterone-lowering treatment, previous convictions, number and age of victims, SSPI-score (Sexual screening scale for pedophilic interest, [44]), and consumption of child exploitation material. Otherwise, three out of four ISIC with high values of sexual arousal were diagnosed with paraphilia (pedophilia, ICD-10, F65.4, [30]), but only seven out of 27 ISIC with low values. This was, however, only evident at a trend level: *χ*^*2*^(1) = 3.372, *p* = 0.066 (see Table 4). Note, that this difference was not seen for the ISOCFP.

With respect to other ICD-10 diagnoses, no differences were found in the ISOCFP, IOFP, but for the ISIC. Three out of four ISIC with high values of *sexual arousal* were diagnosed with any affective disorder (ICD-10, F30–39, depressive disorder), but only 6 out of 26 ISIC with low values of *sexual arousal* (*χ*^*2*^(1) = 4.451, *p* = 0.035). Moreover, there is an overlap in the sense that two out of four ISIC with high sexual arousal values suffer not only from pedophilia but also from affective disorder. Additionally, two ISIC with high values of *sexual arousal* received psychopharmacological (anti-depressive) treatment, but only one out of 27 of the subgroup with low values (*χ*^*2*^(2) = 8.254, *p* = 0.016).

Both ISOCFP with high values of *sexual arousal* also received psychopharmacological (anti-depressive) treatment, while it is only one out of 8 in the subgroup with low values (*χ*^*2*^(1) = 3.750, *p* = 0.053).

**Table 4:** Comparison of participants with low and high values (equal to or above 4) in the “Current-State-of-Emotions-Questionnaire” (CSEQ), (all experiments and both measurements) for the dimension *sexual arousal* per subject group.

	Group	ISOCFP Ss. Low: *n* = 8 Ss. High: *n* = 2		ISIC Ss. Low: *n* = 27 Ss. High: *n* = 4		IOFP Ss. Low: *n* = 7 Ss. High: *n* = 1		IWO Ss. Low: *n* = 164 Ss. High: *n* = 28	
Variable	Subgroups	Ss. low values	Ss. high values	Ss. low values	Ss. high values	Ss. low values	Ss. high values	Ss. low values	Ss. high values
Age	Mean (SD)	40.50 (14.76)	33.50 (7.78)	40.15 (9.68)	41.75 (10.60)	39.29 (10.03)	20	25.20 (5.72)	23.79 (4.32)
	Statistics^f^	*U* = 7.000, *p* = 0.889		*U* = 60.500 *p* = 0.712		*U* = .000 *p* = 0.250		*U* = 1934.500, *p* = 0.182	
Intelligence^a^	Mean (SD)	76.63 (12.70)	84.50 (26.16)	96.16 (18.94)	95.50 (24.17)	92.43 (14.42)	76.00	115.56 (13.64)	111.64 (14.91)
	Statistics^f^	*U* = 10.000, *p* = 0.711		*U* = 50.500, *p* = 1.000		*U* = .000 *p* = 0.250		*U* = 1935.000, *p* = 0.184	
Current sexual activity^b^	Statistics^g^	*χ*^*2*^(2) = 1.667, *p* = 0.435		*χ*^*2*^(4) = 1.008, *p* = 0.909		*χ*^*2*^(2) = .381, *p* = 0.827		*χ*^*2*^(4) = 4.233, *p* = 0.375	
Current relationship status^c^	Statistics^g^	*χ*^*2*^(1) = .278, *p* = 0.598		*χ*^*2*^(4) = 2.187, *p* = 0.701		*χ*^*2*^(1) = .163, *p* = 0.686		*χ*^*2*^(3) = .940, *p* = 0.816	
Pedophilia (ICD-10)	*n* Ss.	4/8 50%	1/2 50%	7/27 28%	3/4 75%	n.a.		n.a.	
	Statistics^g^	*χ*^*2*^(1) = 0.0, *p* = 1.000		*χ*^*2*^(1) = 3.372, *p* = 0.066					
Affective disorder (ICD-10, F30-F39)^d^	*n* Ss.	1/8 12.5%	0/2 0%	6/27 23.1%	3/4 75%	0/7 0%	0/1 0%	n.a.	
	Statistics^g^	*χ*^*2*^(1) = .278, *p* = 0.598		*χ*^*2*^(1) = 4.451, *p* = 0.035		n. a.			
Current psychopharmacological treatment	*n* Ss.	2/8 25%	2/2 100%	1/27 3.8%	2/4 50%	5/7 71.4%	1/1 100%	n.a.	
	Statistics^g^	*χ*^*2*^(1) = 3.750, *p* = 0.053		*χ*^*2*^(2) = 8.254, *p* = 0.016		*χ*^*2*^(1) = .381, *p* = 0.537			
Testosterone-lowering treatment	*n* Ss.	1/8 12.5%	1/2 50%	0/27 (0%)	0/4 (0%)	0/7 (0%)	0/1 (0%)	n.a.	
	Statistics^g^	*χ*^*2*^(1) = 1.406, *p* = 0.236		n.a.		n.a.			
Previous convictions Mean (SD)	Mean (SD)	2.38 (2.07)	1.00 (0.00)	.80 (1.29)	.25 (.50)	8.29 (8.08)	7.00	n.a.	
	Statistics^f^	*U* = 5.000*, p* = 0.533		*U* = 40.000, *p* = 0.562		n.a.			
Number of victims Mean (SD)^e^	*n* Ss.	7.38 (5.5)	8.00 (5.66)	9.67 (28.46)	6.50 (7.78)	n.a.		n.a.	
	Statistics^f^	*U* = 9.000, *p* = 1.000		*U* = 14.500, *p* = 0.659					
Age of victims Mean (SD)^e^	Mean (SD)	8.83 (2.11)	7.38 (1.94)	9.58 (3.47)	11.17 (3.02)	n.a.		n.a.	
	Statistics^f^	*U* = 4.000 *p* = 0.643		*U* = 14.000, *p* = 0.641					
SSPI Mean (SD)^e^	Mean (SD)	4.13 (0.99)	4.00 (1.41)	2.91 (1.04)	3.50 (0.71)	n.a.		n.a.	
	Statistics^f^	*U* = 7.500 *p* = 0.889		*U* = 15.500, *p* = 0.410					
Consumption of child exploitation material	*n* Ss.	n.a.		22/27 81.5%	4/4 100%	n.a.		n.a.	
	Statistics^f^			*χ*^*2*^(1) = 0.883, *p* = 0.347					

*ISOCFP* Individuals who have committed sexual offenses against children and have been placed in forensic psychiatric facilities, *ISIC* Individuals with a self-reported sexual interest in children without being placed in forensic psychiatric facilities, *IOFP* Individuals who have committed other offenses and have been placed in forensic psychiatric facilities, *IWO* Individuals without sexual interest in children and without offense histories.

*n* Number of subjects, *SD* Standard deviation, *n.a*. not applicable, *Ss* Low Subjects with low values (below 4) in the “Current-State-of-Emotions-Questionnaire” (CSEQ), *Ss* High Subjects with high values (equal to or above 4) in the “Current-State-of-Emotions-Questionnaire” (CSEQ).

^a^Intelligence was assessed by the Wechsler Adult Intelligence Scale (27) or Raven’s Progressive Matrices (28).

^b^Current sexual activity was scored with: partnership with sexual intercourse, partnership without sexual intercourse, no partnership and frequent sexual intercourse, no partnership and no sexual intercourse, never had sexual intercourse.

^c^Current relationship status was scored with: single, partnership, married, divorced/legally separated, widowed.

^d^With respect to any ICD-10 diagnoses, only data for affective disorders are presented. Subjects with low and high values regarding sexual arousal did not differ with respect to any other ICD-10 diagnosis.

^e^Criminological variables Number and age of victims are reported only for the ISOCFP (*n* = 10) and ISIC with at least one sexually abused child (*n* = 14). One ISIC reported about 100 victims. Age of victims was available for eight ISOCFP and 13 ISIC. SSPI: Screening Scale for pedophilic interest (44), SSPI-Score could be computed for 10 ISOCFP and 13 ISIC. Information on consumption of child exploitation material was only available for ISIC. For more details, please see section: Section Participants.

^f^Test statistics: non-parametric Mann-Whitney test for independent samples.

^g^Test statistics: Chi-square test for independent samples.

## Discussion

During the conduct of our experiments aiming at the measurement of deviant sexual interest, we observed high subjective sexual arousal in few participants while being confronted with sexual images. Therefore, in this exploratory retrospective data analysis, we were interested in the impact of sexual images on self-reported subjective sexual arousal before and after our experiments.

Our sexual stimuli elicit a moderate mean increase in subjective sexual arousal. This moderate increase of subjective *sexual arousal* is an expected and intended effect when someone is confronted with sexual stimuli, and is also in line with the known heightened subjective sexual arousal after being confronted with sexual stimuli (e.g., [45–47]). But, looking at individual cases, up to 14.3% of participants indicated high sexual arousal values before, but mainly after the experiments. ISIC, ISOCFP, and IOFP among those individuals with high sexual arousal values were more likely to be diagnosed with paraphilia (pedophilia) and/or affective disorders, and to receive anti-depressive treatment.

### Individual participants, who feel highly sexually aroused when presented with sexual stimuli, are “special”

The individual analysis demonstrated up to 20% high values per group, which should be of importance in the context of our research field. Contrary to previous findings [48, 49], participants with high or low levels of subjective sexual arousal do not differ regarding a large number of demographic, clinical, and criminological variables. Extant literature indicated that individuals, who have committed sexual offenses, have lower intellectual abilities (e.g., [48, 49]). Moreover, lower intellectual abilities have been associated with riskier (sexual) behavior, which itself is more pronounced in states of high sexual arousal [12]. Supporting this research, the lowest IQ in our sample was seen for the ISOCFP, in which also the highest proportion of participants with high subjective arousal values is to be found. Otherwise, within this group, the IQ does not seem to have an impact on the level of experienced sexual arousal. The same holds true for our other subject groups, with rather close to normal IQ-scores. Similarly, contrary to other studies [16, 17], participants with higher subjective arousal levels do not seem to be more delinquent than participants with lower values. Hence, there could be factors other than intellectual ability and delinquency which may have impacted the experienced sexual arousal level.

Indeed, some clinical factors seem to play a role, such as psychiatric diagnoses and pharmacological treatment. The finding that highly sexually aroused ISIC were more likely to be diagnosed with pedophilia is in line with a large amount of research, which repeatedly demonstrated that sexual interest in children is one of the most important risk factors for (sexual) recidivism (e.g., [22]). A diagnosis of pedophilia could be associated with higher subjective sexual arousal when being confronted with sexual stimuli, which could in turn have an impact on the risk of offending. The higher number of ISIC with depressive disorders and anti-depressive treatment among those with high values of experienced sexual arousal seems to be rather contra-intuitive. There is a well-established relationship between depression and anti-depressive treatment on the one hand and sexual dysfunction and decreased sexual arousal on the other hand (e.g., [50, 51]). Otherwise, even though not unchallenged, a relationship between depressive symptoms, affective instability, and maladaptive emotion regulation strategies has been discussed for a long time (e.g., [50, 52]). Additionally, deficits in emotion regulation are a well-known and important risk factor for violent and sexual offending [53, 54]. Hence, it can be speculated that our ISIC with depressive disorders could have felt strong sexual arousal while being unable to suppress or regulate it successfully. In two of those ISIC the strong sexual arousal might be associated with their pedophilic disorder. Affective disorders are among the known psychiatric comorbidities in individuals with pedophilia [48]. One can speculate that in these two ISIC the co-occurrence of a paraphilic disorder with stronger deviant sexual interest and the affective disorder with affective instability, and maladaptive emotion regulation strategies could have had a synergistic effect on their experienced higher subjective sexual arousal.

Two non-depressive ISOCFP with high experienced sexual arousal values also received anti-depressive medications (SSRIs, selective serotonin reuptake inhibitors), but with the aim to decrease sexual functioning. At a speculative level, side effects of SSRIs, such as “feeling agitated”, together with diagnosed personality disorder with emotionally unstable components could have led to these strong feelings in the two ISOCFP. Therefore, the participants’ type of psychiatric disease and its degree of severity indeed seems to be an important fact to consider as we were able to observe an acute exacerbation of symptoms in one of these two ISOCFP after study participation.

### How should we continue with the research project?

We have shown that the confrontation with sexual stimuli in our laboratory studies is associated with high self-reported sexual arousal in up to 14.3% of participants. Among IWO, up to 12% felt highly sexually aroused based. Of course, we do not expect healthy participants to commit sexual crimes due to high sexual arousal induced by treatment participation. We assume that a combination of high subjective sexual arousal while being confronted with sexual stimuli and personal factors, such as criminal history, impulsivity, mental disorder, and emotion-regulation abilities might modulate the individual risk of committing sexual crimes. Hence, the confrontation with sexual images might have increased the risk for (sexual) recidivism in some of our ISIC and ISOCFP with high sexual arousal.

How can we face these challenges under consideration of costs and benefits in this special forensic research field? Obviously, individuals with high sexual arousal values after the confrontation with sexual stimuli do not necessarily commit sexual crimes. Despite the association between sexual arousal and sexual offending, sexual arousal measures cannot solely be used to predict sexual recidivism. It is assumed that most individuals who are sexually attracted to children have never acted upon their sexual interest [55, 56]. In our data analysis, a heightened sexual arousal was not associated with single criminological variables such as number of victims or pre-convictions. This, however, does not mean that an association between those factors does not exist.

Concerning our research project, we decided to interrupt any further examination of individuals with sexual interest in children and individuals who have committed offenses. With respect to the small but important proportion of participants with a strong increase of experienced sexual arousal, we are now developing a detailed risk management concept which extends the present clinical and security evaluations, inclusion -, and exclusion criteria. This includes comprehensive clinical diagnostics focusing on affective disorders and sexual deviance, a more detailed individual risk assessment, and questionnaires assessing affective lability and emotional regulation abilities for instance.

### Limitations

This data analysis has several limitations. Most importantly, the main objective of our larger research project was the measurement of sexual interest, but not the characterization of individual sexual arousal. For this exploratory analysis of changes in feelings following the presentation of sexual images, we used our self-developed CSEQ [6, 7], for which test validity has not been established. We only included one item to ask for sexual arousal, which can serve as a first approach, but not as a reliable measurement of sexual arousal. Furthermore, we only observed associations between sexual stimulus presentation and partially high subjective sexual arousal levels in our participants. A causal relationship remains to be examined. Another important limitation considers the assessment of sexual arousal via subjective self-report. It is well-known that self-reports are susceptible to manipulations, which is of special relevance in the forensic context [57]. However, assuming that participants tended to give socially desirable answers and rather understated their experienced sexual arousal, the real sexual arousal could be higher than we found. Furthermore, some participants reported high sexual arousal already in the pre-measurements. It might be that those high sexual arousal levels in the pre-measurements are unrelated to the experiments. Nonetheless it is also possible that the individual expectation to see sexual images could have increased subjective sexual arousal levels already before the experiments. For this first exploratory analysis we wanted to report raw data without any data exclusions and baseline-transformations. Notwithstanding, future studies should additionally analyze individual decreases and increases of subjective sexual arousal levels to better characterize the extent of changes. Moreover, participants with high sexual arousal level in pre-measurements should receive more attention in order to investigate potential reasons for this high sexual arousal. Another critical aspect concerns the low number of participants in both ISOCFP and IOFP. Although the proportion of participants with high sexual arousal values was similar in all groups, only one to two individuals of those groups were highly sexually aroused. Hence, the significance of these results is very limited. Moreover, one could argue that the occurrence of extreme values in empirical research is absolutely normal. Hence, the question arises of whether those values should be excluded from the data analysis [58]. In our view, every single participant with high self-reported sexual arousal in association with the confrontation with sexual stimuli should be identified in order to recognize vulnerable individuals. Thus, in order to select those individuals with high values of subjective sexual arousal, we used this rather artificial value of “equal to or above 4 on our 9-point Likert scale” for our explorative data analysis. Furthermore, it still needs to be investigated, whether such high subjective sexual arousal scores actually reflect potentially risky sexual behavior. One approach could be to examine associations between subjective sexual arousal levels and acute dynamic risk concepts, such as “sexual preoccupation” and “emotional collapse” which are included in ACUTE-2007 [59], a well-known empirical-actuarial risk assessment tool.

Another aspect concerns differences in our sample with respect to several demographic and clinical variables (see Table 1). As can be seen from Table 1, IWO were significantly younger than all other participants. It is known that sexual arousability can decline with age [60]. Additional correlation analyses yielded significant associations between participant age and subjective sexual arousal values in the CSEQ for two of our six dependent variables (three experiments with two measurements each), with decreasing sexual arousal values with increasing age (see Table S2). But these associations do not seem to have an impact on our group differences: Further analyses failed to demonstrate statistically significant group differences regarding subjective sexual arousal values (see table S3). We also did not find a lower percentage of participants with high sexual arousal levels among ISIC, ISOCFP and IOFP compared to IWO (see results section). Therefore, we assume that the age of our participants does not have a significant impact on our results, even if we cannot exclude it. Furthermore, participants differ, for instance, with respect to intelligence and educational level (Table 1). Although estimates of prevalence of intellectual disabilities among persons with offense histories varied widely, the view is accepted that people with intellectual disabilities are overrepresented among persons with sexual-offense histories [61]. As mentioned above, among others, lower intellectual abilities are proposed to be associated with riskier (sexual) behavior [12]. Even though, groups did not differ with respect to mean sexual arousal levels, lower intellectual abilities in patient groups could have had an impact on potential riskier (sexual) behavior in those groups. A last aspect concerns group differences with respect to psychiatric disorders and corresponding medication. The prevalence of sexual dysfunctions is higher in persons with mental disorders, particularly those who are additionally psychopharmacologically medicated (e.g., [62, 63]). Testosterone-lowering medication lead, among others, to reduced sexual arousal [64]. Accordingly, one would expect a lower sexual arousal in those individuals with (medicated) psychiatric disorders (i.e., ISOCFP, ISIC, IOFP) and testosterone-lowering medication (ISOCFP) compared to IWO. But ISOCFP, ISIC, and IOFP with or without any psychopharmacological or testosterone-lowering medication did not differ with respect to subjective sexual arousal (see table S4, S5). Hence, even though an impact of psychiatric disorders and psychopharmacological/testosterone-lowering medications on subjective sexual arousal cannot be excluded, we did not find this in our sample.

### Outlook

Of course, our results have to be replicated with larger groups, more detailed measures of subjective *sexual arousal* (e.g., [45, 65]), and instruments assessing emotion regulation ability (e.g., [66, 67]). Further analyses also have to examine if rather objective eye movement data are associated with subjective measures of sexual arousal. Based on theoretical models (e.g., the information processing approach by Spiering and Everaerd, [68]) we would expect, for instance, a positive association between fixation time on sexual stimuli and subjective sexual arousal level. Otherwise, it is known that subjective experience of sexual arousal and physiological (e.g., genital) measures do not always correspond [47]. So, one question is, if analyses of rather “objective” eye movement parameters could add some more information about the impact of sexual stimuli on sexual arousal levels in our participants.

## Conclusion

By means of this first explorative data analysis, we want to draw attention to the possibility that few individuals may react strongly in association with the confrontation with sexual stimuli, which could be potentially risky. This is important insofar as some of our study participants with high arousal levels are individuals who have committed sexual offenses. We cannot exclude that the confrontation with sexual images might have increased the risk of committing sexual crimes in those individuals. Otherwise, it is not our intention to discourage people from research concerning the measurement of deviant sexual interest, or using established tools to assess deviant sexual interest e.g., the “Explicit and Implicit Sexual Interest Profile” (EISIP, [69, 70]). Instead, we want to emphasize the need to weigh up anticipated scientific knowledge gain and a, albeit rare but potentially dangerous increase of risk, or decompensation due to sexual stimulus confrontation in vulnerable participant groups.

## Supplementary information

Table S1, S2. S3, S4, S5

## Data Availability

The datasets generated for this analysis will not be made publicly available. Our data are highly sensitive (deviant sexual interest/ child sexual offenses) and cannot be anonymized.
