# Supplementary material for: Rare, but potentially risky, high individual increase of self-reported sexual arousal in men, who have committed sexual offenses against children, while being confronted with experimental sexual stimuli — a retrospective data analysis
Source: Int J Impot Res. 2023 Dec 12;37(3):238–50. doi: 10.1038/s41443-023-00802-5 (PMC11981933; doi:10.1038/s41443-023-00802-5)
Supplement: Supplementary file 1 — Table S1, S2. S3, S4, S5 [file 41443_2023_802_MOESM1_ESM.docx]

Table S1. Comparison of subjects with low and high values (equal to or above 4) in the “Current-State-of-Emotions-Questionnaire” (CSEQ) (all experiments and both measurements) for the dimension *Sexual Arousal.*

| **Dimension** | **Group** | **Age**  **Mean (SD)** | **Intelligence**  **Men (SD)^1^** | **Current sexual activity^2^** |
| --- | --- | --- | --- | --- |
| **Sexual arousal** | **Participants with low Values**  ***n* = 206** | 28.23 (9.19) | 110.86 (17.49) | *χ^2^*(4) = 3.264,  *p* = .515 |
|  | **Participants with high Values *n* = 35** | 26.29 (8.00) | 107.23 (18.43) |  |
|  | **Statistics^3^** | *U* = 3062.5  *p* = .154 | *U* = 3142.0  *p =* .257 |  |

^1^ Intelligence was assessed by the Wechsler Adult Intelligence Scale (27) or Raven's Progressive Matrices (28).

^2^ Current sexual activity was scored with:
partnership with sexual intercourse, partnership without sexual intercourse, no partnership and frequent sexual intercourse, no partnership and no sexual intercourse, never had sexual intercourse

^3^ Statistics: non-parametric Mann-Whitney-U-Test for independent for variables age and intelligence. Chi-square test for variable current sexual activity

Abbreviations: *n* = Number of participants, SD: Standard deviation

Table S2. Associations between age of participants and subjective sexual arousal values in the “Current-State-of-Emotions-Questionnaire” (CSEQ).

| Experiment | Initial orientation | | Sexual distractor task | | Stimulus Rating | |
| --- | --- | --- | --- | --- | --- | --- |
|  | Pre-Measurement | Post-Measurement | Pre-Measurement | Post-Measurement | Pre-Measurement | Post-Measurement |
| Statistics^2^ | *r* = -.125  *p* = .251 | *r* = .023  *p* = .838 | *r* = -.091  *p* = .164 | *r* = -.150  *p* = .020 | *r* = -.128  *p* = .049 | *r* = -.085  *p* = .194 |
| Number of participants | 85 | 85 | 236 | 236 | 238 | 238 |

^2^ Statistics: Spearman-Rho Correlation coefficient

Table S3. Group differences with respect to subjective sexual arousal values in the “Current-State-of-Emotions-Questionnaire” (CSEQ). Shown are statistical analyses for each experiment with pre- and post-Measurements

| Experiment | Initial orientation | | Sexual distractor task | | Stimulus Rating | |
| --- | --- | --- | --- | --- | --- | --- |
|  | Pre-Measurement | Post-Measurement | Pre-Measurement | Post-Measurement | Pre-Measurement | Post-Measurement |
| Statistics^2^ | *H*(3) = 4.676  *p* = .197 | *H*(3) = .173  *p* = .982 | *H*(3) = 7.936  *p* = .047^3^ | *H*(3) = 4.120  *p* = .249 | *H*(3) = 1.731  *p* = .630 | *H*(3) = 1.004  *p* = .800 |
| Number of participants | 85 | 85 | 236 | 236 | 238 | 238 |

^2^ Statistics: non-parametric Kruskal-Wallis test for independent samples
^3^ Sexual distractor task, Pre-Measurement: pairwise comparisons: IWO > ISIC, *p* =.053

Abbreviations: ISOCFP: Individuals who have committed sexual offenses against children and have been placed in forensic psychiatric facilities; ISIC: Individuals with a self-reported sexual interest in children without being placed in forensic psychiatric facilities; IOFP: Individuals who have committed other offenses and have been placed in forensic psychiatric facilities; IWO: Individuals without sexual interest in children and without offense histories

Table S4. Group differences with respect to current psychopharmacological medication and subjective sexual arousal values in the “Current-State-of-Emotions-Questionnaire” (CSEQ). Shown are statistical analyses for each subject group and each experiment with pre- and post-Measurements

ISOCFP (*n* =10: 4 individuals with psychopharmacological medications and 6 without)

| Experiment | Initial orientation | | Sexual distractor task | | Stimulus Rating | |
| --- | --- | --- | --- | --- | --- | --- |
|  | Pre-Measurement | Post-Measurement | Pre-Measurement | Post-Measurement | Pre-Measurement | Post-Measurement |
| Statistics^2^ | *U* = 9.000  *p* = .610 | *U* = 5.000  *p* = .171 | *U* = 9.000  *p* = .610 | *U* = 4.500  *p* = .114 | *U* = 4.000  *p* = .114 | *U* = 4.500  *p* = .114 |

^2^ Statistics: non-parametric Mann-Whitney-U-Test for independent samples

ISIC (*n* = 31: 4 individuals with psychopharmacological medications and 27 without)

| Experiment | Initial orientation | | Sexual distractor task | | Stimulus Rating | |
| --- | --- | --- | --- | --- | --- | --- |
|  | Pre-Measurement | Post-Measurement | Pre-Measurement | Post-Measurement | Pre-Measurement | Post-Measurement |
| Statistics^2^ | *U* = 56.00  *p* = .737 | *U* = 22.00  *p* = .082 | *U* = 39.000  *p* = .944 | *U* = 29.00  *p* = .635 | *U* = 32.000  *p* = .799 | *U* = 22.500  *p* = .313 |

^2^ Statistics: non-parametric Mann-Whitney-U-Test for independent samples

IOFP (*n* = 8: 6 individuals with psychopharmacological medications and 2 without)

| Experiment | Initial orientation | | Sexual distractor task | | Stimulus Rating | |
| --- | --- | --- | --- | --- | --- | --- |
|  | Pre-Measurement | Post-Measurement | Pre-Measurement | Post-Measurement | Pre-Measurement | Post-Measurement |
| Statistics^2^ | *U* = 6.000  *p* = 1.000 | *U* = 2.000  *p* = .381 | *U* = 5.000  *p* = .857 | *U* = 4.00  *p* = .643 | *U* = 5.000  *p* = .857 | *U* = 3.000  *p* = .429 |

^2^ Statistics: non-parametric Mann-Whitney-U-Test for independent samples

Abbreviations: ISOCFP: Individuals who have committed sexual offenses against children and have been placed in forensic psychiatric facilities; ISIC: Individuals with a self-reported sexual interest in children without being placed in forensic psychiatric facilities; IOFP: Individuals who have committed other offenses and have been placed in forensic psychiatric facilities; IWO: Individuals without sexual interest in children and without offense histories

Table S5. Group differences with respect to current testosterone-lowering medication and subjective sexual arousal values in the “Current-State-of-Emotions-Questionnaire” (CSEQ). Shown are statistical analyses for each experiment with pre- and post-Measurements

ISOCFP (*n* = 10: 2 individuals with testosterone-lowering medication and 8 without)

| Experiment | Initial orientation | | Sexual distractor task | | Stimulus Rating | |
| --- | --- | --- | --- | --- | --- | --- |
|  | Pre-Measurement | Post-Measurement | Pre-Measurement | Post-Measurement | Pre-Measurement | Post-Measurement |
| Statistics^2^ | *U* = 9.000  *p* = 1.000 | *U* = 3.000  *p* = .267 | *U* = 9.000  *p* = 1.000 | *U* = 5.500  *p* = .533 | *U* = 6.000  *p* = .711 | *U* = 5.500  *p* = .533 |

^2^ Statistics: non-parametric Mann-Whitney-U-Test for independent samples

Abbreviations: ISOCFP: Individuals who have committed sexual offenses against children and have been placed in forensic psychiatric facilities; ISIC: Individuals with a self-reported sexual interest in children without being placed in forensic psychiatric facilities; IOFP: Individuals who have committed other offenses and have been placed in forensic psychiatric facilities; IWO: Individuals without sexual interest in children and without offense histories
